# Proline Rich Motifs as Drug Targets in Immune Mediated Disorders

**DOI:** 10.1155/2012/634769

**Published:** 2012-05-16

**Authors:** Mythily Srinivasan, A. Keith Dunker

**Affiliations:** ^1^Department of Oral Pathology, Medicine and Radiology, Indiana University School of Dentistry, Indiana University Purdue University at Indianapolis 1121 West Michigan Street, DS290, Indianapolis, IN 46268, USA; ^2^Department of Biochemistry and Molecular Biology and School of Informatics, Indiana University School of Medicine, Indiana University Purdue University at Indianapolis, Indianapolis, IN, USA

## Abstract

The current version of the human immunome network consists of nearly 1400 interactions involving approximately 600 proteins. Intermolecular interactions mediated by proline-rich motifs (PRMs) are observed in many facets of the immune response. The proline-rich regions are known to preferentially adopt a polyproline type II helical conformation, an extended structure that facilitates transient intermolecular interactions such as signal transduction, antigen recognition, cell-cell communication and cytoskeletal organization. The propensity of both the side chain and the backbone carbonyls of the polyproline type II helix to participate in the interface interaction makes it an excellent recognition motif. An advantage of such distinct chemical features is that the interactions can be discriminatory even in the absence of high affinities. Indeed, the immune response is mediated by well-orchestrated low-affinity short-duration intermolecular interactions. The proline-rich regions are predominantly localized in the solvent-exposed regions such as the loops, intrinsically disordered regions, or between domains that constitute the intermolecular interface. Peptide mimics of the PRM have been suggested as potential antagonists of intermolecular interactions. In this paper, we discuss novel PRM-mediated interactions in the human immunome that potentially serve as attractive targets for immunomodulation and drug development for inflammatory and autoimmune pathologies.

## 1. Protein-Protein Interactions

Protein-protein interactions (PPIs) are critical for most biological functions and cellular processes [1, 2]. Under appropriate environmental conditions, the PPIs take place through an interface governed by shape, chemical complementarity, and flexibility of the interacting molecules. Different types of PPIs have been described. Homo- or heterologous oligomeric PPI complexes represent isologous or heterologous association of identical protein units. PPI complexes of interdependent protomer units are referred to as obligate complexes as opposed to nonobligate complexes that occur independently [3, 4]. The strength of PPI is represented by the dissociation constant (K_D_) expressed in molar concentration and derived from the ratio between the dissociation and association rate constants. Based on duration and affinity, PPIs can be classified as strong interactions that exhibit K_D_ values with *μ*M concentrations and weak or transient interactions with values in the mM or higher concentrations. Transient PPIs are further divided into strong and weak transient interactions. While strong transient PPIs require a molecular trigger such as ligand binding to shift the oligomeric equilibrium, weak transient interactions are mediated by binding between a few critical residues [3, 5]. 

Traditionally, PPIs are thought to be mediated by “lock and key” or “induced-fit” interaction between large structured domains [6]. However, characterization of increasing numbers of protein sequences and structures has suggested that the interacting modules of multidomain proteins can be distinguished as globular domains, as short peptide functional sites, and as long peptides that interact with their partners over extensive regions. Thus, three distinct protein-protein interfaces are recognized [7]. While the evolutionarily conserved domain-domain interfaces are large and relatively stable, the evolutionarily plastic domain peptide interfaces are smaller and transient [8–10]. Protein interaction domains can be classified based on sequence homology, ligand-binding properties, or structural similarity [11]. Thus, a typical class of ligand binding proteins may contain a variety of protein interaction domains that recognize a common ligand, whereas a family classified based on sequence homology contains a single fold that may recognize a variety of ligands. Some families function in a narrow cellular context, while others participate in a diverse range of processes [11, 12].

## 2. Protein:Peptide Interactions: Linear Motifs and Molecular Recognition Features (MoRFs)

Interactions between globular proteins or domains and peptide have been investigated in parallel using biological data and/or computational methods [7, 13–19]. Short segments of structured binding sites within long disordered regions of proteins are referred to as linear motifs (LMs) or as molecular recognition features (MoRFs) [13–15, 18]. While LMs are identified/predicted by sequence patterns, MoRFs are identified by sequence features associated with disorder prediction [13, 14, 17, 18].

A systematic survey of LM-mediated protein interactions estimated that 15–40% of all interactions in a typical eukaryotic cell are mediated through protein-peptide interactions [20]. Predictions over nine proteomes of MoRFs that often form *α*-helices upon binding indicate that about 44 ± 4% of eukaryotic proteins contain potential helix-forming MoRFs [21]. These LM and MoRF frequency estimates concur in the suggestion that a large fraction of macromolecular complexes is affected either directly or indirectly by peptide-binding events. The protein interface is predefined and ready to accommodate the binding peptide. For efficient interaction, the peptide “scans” the protein surface for a large enough pocket into which it anchors through a small number of residues or core motif that contribute maximally to the free energy of binding [22]. The hot spot residues show a tendency to be localized in the center, and the number of hot spots is partially dependent on the length of the interacting motif [14, 23]. In general, the average solvent-accessible surface area that is buried upon peptide binding is less than half the area buried in protein-protein complexes. Furthermore, the peptides tend to bind in a more planar fashion, optimize hydrogen bonds, and display better packing than proteins at the interface [10, 20].

## 3. Amino Acid Propensities in LMs

In terms of amino acid composition, the sequence of LMs can be distinguished into typical patterns. Each LM possesses a set of conserved residues having restricted identities that serve as specificity determinants and a second set of fully variable residues that likely act as spacers [14, 24]. The spacers and flanking regions exhibit preferential presence of disorder promoting charged residues. The restricted identities of LM are enriched in proline as well as in hydrophobic residues including phenylalanine, leucine, tryptophan, tyrosine, and isoleucine [14, 25]. The relative paucity of glycine and alanine in the LMs may represent strategies to simultaneously curb excessive flexibility and restrict the tendency to form strong secondary structural elements [25–27]. Systematic analyses suggested that, as compared to the general disordered regions, MoRFs are also enriched in hydrophobic residues, in particular the aromatic residues [15, 18, 25]. In many cases, LMs and MoRFs identify the same region of sequence. The function of LMs is essentially embodied in the primary amino acid sequence independent of tertiary structure and is strongly context dependent which defines the natural constraints that act on these motifs. Within protein structures, LMs are predominantly observed in the solvent-exposed regions such as the loops, in intrinsically disordered regions or between defined domains [28].

## 4. Significance of Proline in LMs

Of special significance is the preponderance of proline both in the conserved identities and in the flanking regions of LMs [24, 25]. Among the naturally occurring amino acids, proline is unique in several features. It is the only residue with substituted amide nitrogen. Proteins that recognize the *δ* carbon on the substituted amide nitrogen within the context of the otherwise standard peptide backbone can select precisely for proline at a given position without making extended contacts with the rest of the side chain [29]. This facilitates sequence-specific recognition without requiring a particularly high-affinity interaction [30]. Such specific and weak bindings are important for cellular communication and signaling functions that require rapidly reversible interactions [31]. 

Proline is the only naturally occurring amino acid in which the side chain atoms form a pyrrolidine ring with the backbone atoms. This cyclic structure mediates the slow isomerization between cis/trans conformations [32]. The polyproline stretches can adopt two unique helical conformations, I and II [33]. Polyproline type I (PP_I_) is a right-handed helix consisting of cis-prolines. While poly-L proline in apolar solvents can adopt the PP_I_ conformation, there is paucity of PP_I_ helical segments in proteins [33, 34]. PP_II_ helix is a left-handed helix, consists of proline in trans-conformation, but also accommodates frequently other amino acids such as glutamine, serine, and arginine [35, 36]. With three residues per turn, the PP_II_ helix is an extended structure and has an overall shape resembling a triangular prism. PP_II_ helices are widely distributed in the eukaryotic proteome and hence are of greater biological significance [37, 38]. 

The unusual shape of the proline side chain imposes structural constraints on adjacent residues such that the proline rich motif (PRM) preferentially adopts the left-handed PP_II_ helical conformation [37, 39]. In PP_II_ helix, both the side chains and the backbone carbonyls point out from the helical axis into solution at regular intervals [40]. Furthermore, the lack of intramolecular hydrogen bonds primarily due to the absence of a backbone hydrogen-bond donor on proline leaves these carbonyls free to participate in intermolecular hydrogen bonds. Thus, both side chains and carbonyls can easily be “read” by interacting proteins making PP_II_ helix an excellent recognition motif [37]. In addition, since the backbone conformation is already restricted, the entropic cost of binding is reduced [41]. In contrast to the enthalpy-induced associations such as the lock and key model, PP_II_ helices are entropy driven and behave as “adaptable gloves” in order to obtain the correct recognition. Indeed, in a recent study that reported significantly lower configurational entropy for known peptide inhibitors, polyproline peptides were among those with lowest entropy values [42]. While the intrinsic properties of the proline facilitate the PP_II_ helix formation, the conformation is potentially stabilized by the surrounding water molecules supporting the preponderance of PRM in solvent exposed loops/disordered regions of proteins [37–39]. Furthermore, it has been observed that, in addition to the enrichment of proline and hydrophobic residues, the LMs are also rich in charged residues including arginine and aspartic acid [24, 25]. Positively charged residues both local and nonlocal to the PP_II_ helices satisfy the H-bond donor potential of the main-chain carbonyls and stabilize the PP_II_ conformation [43]. An advantage of focusing on such distinct chemical features is that such interactions can be discriminatory without resorting to extremely high affinities [37]. Indeed, PRM-mediated interactions exhibit fast on and off rates of binding adopted for effective control and regulatory functions [31, 38].

## 5. Nature of PRM-Binding Domain (PRD)

The binding between the PRM and the protein domains relies on interactions with core-flanking epitopes of the motif and the PRD interface residues to achieve the necessary specificities [14, 25, 44]. The amino and carboxyl termini of the PRD are generally located relatively close together allowing the domains to slot into their respective host proteins with minimal disruption of the overall protein structure [45]. Structurally, the PRD themselves are found in exposed and accessible regions to recruit target proteins. The PRDs are enriched in aromatic residues that often selectively interact with the critical proline of the PRM in the binding interface. The planar structure of the aromatic side chains appears to be highly complementary to the ridges and grooves presented on the PP_II_ helix formed by the PRM [45, 46]. 

Summarizing, if a recognition event involves the distinctive property of proline among the 20 natural amino acids, the interaction does not have to be of particularly high affinity to be selective [47]. The benefits of weak, but specific, interactions in intracellular signaling pathways may help explain the preponderance of proline-based recognition motifs in the eukaryotic proteome [48]. Indeed, a recent survey revealed an abundance of the polyproline motif “PXXP” in various gene ontology groups of proteins including enzymes, cytoskeletal proteins, nucleic acid-binding proteins, transport proteins, splicing factors, metal-binding proteins, and ribosomal proteins suggesting an evolutionary conservation of protein-protein networks centered on PRMs [49].

## 6. PRD:PRM Interactions in the Immunie Responses

The human immunome network elicited so far consists of nearly 1400 interactions involving approximately 600 proteins [50]. Several protein:peptide interactions have been shown to be pivotal for the formation of molecular assemblies of functional complexes that include membrane bound receptors, cytoplasmic signaling molecules, and transcriptional regulatory proteins [51–53]. The life time of the protein complexes as well as the regulatory processes are tightly controlled for proper functioning [29, 44]. 

PRM-mediated intermolecular interactions are observed in many facets of the immune response including antigen recognition, cell-cell communication, and signaling [46]. Stimulation of lymphocytes with a specific antigen initiates a cascade of signal transduction events that are integrated by numerous adapter proteins which function to establish larger protein complexes and promote complete activation. Many of these adapter proteins possess specific protein domains such as the Src homology 3 (SH3) domains and the WW (named for two tryptophans (W)) domains that selectively recognize proline rich regions in their interacting partners [12, 46]. In addition, many cell surface and intracellular proteins in the immunome exhibit one or two proline-rich regions that interact with highly conserved hydrophobic residues in their binding partners and mediate transient protein-protein interactions [14, 44, 48]. The advantages of transient protein-peptide interactions for functioning of the immune system can be enumerated as follows.

1. The small interface between the peptides and their protein domain partners facilitates low-affinity weak interactions that are easily formed and disrupted to regulate cellular responses. Indeed cell surface receptors that mediate immune responses are often coupled to intracellular signaling pathways by recognition of modular protein interaction domains that bind a short LM for example, CD2:CD2BP interaction (K_D_ = *μ*M) [54].
2. One protein can bind multiple peptides providing an elegant mechanism that uses transient interactions for bringing together different combinations of complexes each with different functions leading to a different signal and response, for example, CD80:CD28/CD152 interactions [55].
3. The low-affinity binding allows for large number of interactions of short durations ranging between 10 s and 100 s decreasing the possibility of sustained adherence and facilitating fleeting contacts critical for cell-cell communications such as the interactions between the antigen presenting cells and the T cells mediated by integrins [56, 57].

In this paper, we discuss novel PRD:PRM interactions that could potentially serve as attractive targets for immunomodulation and drug development for inflammatory and autoimmune pathologies. The paper does not include the widely recognized SH3 domain:peptide interactions. Readers interested in these interactions are referred to the excellent reviews [45, 58].

## 7. PRD:PRM Interactions in Cell Surface Immunome

Proteins located at the surface of immune cells are of particular significance in migration, in specific antigen recognition, in modulating the function of receptors for immune response mediators such as the cytokines as well as in highly focused fine control of intercellular interactions between proteins on opposing cells. Many such interactions have been characterized using monoclonal antibodies [51, 59]. Differential expression and/or function of cell surface proteome in health and disease have been reported in several immune-mediated pathologies substantiating the potential role of select immune cell surface proteins as excellent targets for diagnostic and therapeutic interventions [60].

## 8. T-Cell Costimulatory Receptor: Ligand Interactions

**Figure 1:**
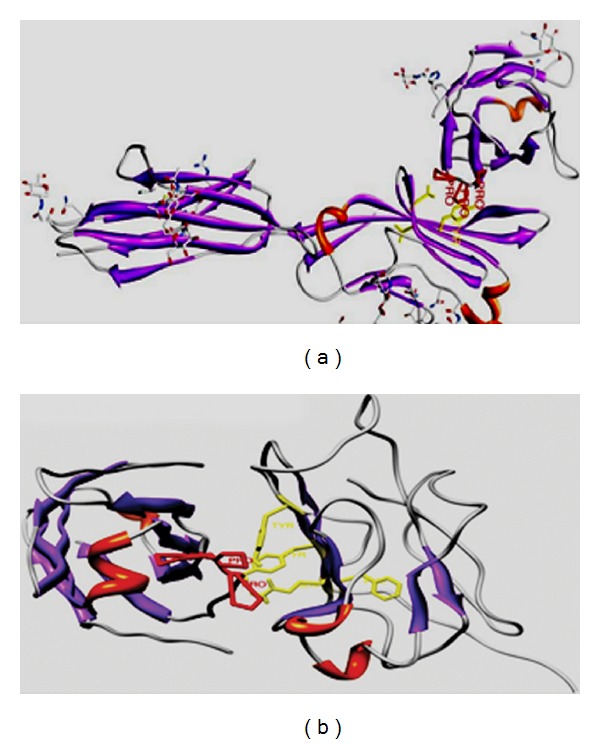
(a) represents the solution structure of the CD152:CD80 complex (PDB1I8 L), and (b) represents the complex of ICOS:ICOS L. Homology modeling of ICOS and ICOSL was predicted using the structures of CD152 (PDB 1AH1 and PDB 1DQT), CD80 (PDB 1DR9) respectively, as templates by Geno 3D [89] and SWISS MODEL [90]. Prediction of the structural complex was performed by ClusPro [91], the complex with least energy is shown.

A manually curated database suggested that over 20% of the human cell surface immunome consists of members of the immunoglobulin superfamily which includes the T-cell costimulatory molecules [61, 62]. In addition to the antigenic stimulation, complete activation of lymphocytes requires costimulatory receptor-ligand interactions that modulate the strength, course, and duration of the immune response [63, 64]. One of the better characterized complexes include the interactions between the costimulatory receptors CD28/CD152 or ICOS expressed on T cells and the CD80/CD86 and ICOSL ligands on the antigen presenting cells. Structurally, the CD28, CD152, and ICOS are composed of a single extracellular IgV domain linked to a stalk region and a transmembrane segment followed by a relatively short cytoplasmic tail, which contains at least one tyrosine-based signaling motif [65]. The receptors exhibit significant homology in primary sequence and share a consensus sequence consisting of three consecutive prolines in the complementarity determining region-3 like region/FG loop [56]. In CD28 and CD152, these prolines are embedded within the MYPPPY sequence, while, in ICOS, they are embedded in the FDPPPF sequence. Mutagenesis experiments indicate that the polyproline motif is essential for the binding of CD152, CD28, and ICOS to their respective ligands [66, 67]. The crystal structures of the murine CD152 bound to CD80 or CD86 reveal that the binding interface is formed predominately by contacts between the MYPPPY sequence of the CD152 FG loop and a concave surface on the front sheet of the CD80 ligand (Figure 1). The three proline residues in the polyproline motif adopt a unique open cis-trans-cis main chain configuration that exhibits geometric complementarity to the binding pocket of the ligand [62, 68]. Secondary structure prediction by PROSS suggests that the second proline of CD152 in the bound complex with CD80 adopts PP_II_ helical conformation with the *ϕ* and Ψ angles of −76 and 164.6, respectively [69, 70]. In the molecular model of ICOS built using the solution structure of CD152 as template, the critical proline that interacts with the ICOSL binding interface exhibits *ϕ* and Ψ angles of −53.4 and 167.4, respectively [71] (Tables 1 and 2; Figure 1). The sequence and structural homology together with functional similarity of the FG loops suggest that these receptors share a common mode of recognition for their ligands. The ectodomains of the CD80, CD86, and ICOSL possess a membrane proximal IgC and a distal IgV domain that makes substantial contact with the solvent exposed polyproline motif in the FG loop of the receptors through a surface with considerable hydrophobic character [71–73]. The extended PP_II_ helical conformation facilitates the backbone atoms of the PRM to form hydrogen bonds with the conserved tyrosine at the CD80/ICOSL interface [30, 37, 68]. Intriguingly, it has been suggested that the presence of phenylalanine, a more hydrophobic and poor hydrogen bond acceptor/donor, in the binding interface of CD86 perhaps contributes to the absence of PP_II_ helical conformation and lower affinity for the interactions between CD152 and CD86 [73]. Thus, the local environment of the CD80 binding pocket and the orientation of the functionally important proline in the PRM of CD152 may account for the difference in the strength of interaction between the CD152 and the CD80/CD86. Knowledge derived from the contact preferences of amino acids at PPI interface and the residue propensity to form PP_II_ helix has been adopted in the design of a small peptide, the CD80-competitive antagonist peptide (CD80-CAP) that mimics the ligand binding conformation of the receptor and inhibits CD28/CD152:CD80 interactions [69] (Figure 1). Treatment with CD80-CAP suppressed T-cell-mediated inflammatory responses in mouse models of rheumatoid arthritis, multiple sclerosis, and inflammatory bowel disease [74–76].

## 9. PRM:PRD Interactions in the Cytoskeleton

Efficient accomplishment of immune responses in inflammation and infection requires a finely regulated cytoskeleton to enable cellular membrane reorganization, receptor localization, and recruitment of signaling molecules, all of which are crucial for immune cell activation, proliferation, secretion, migration, and survival [77]. The cytoskeleton consists of filamentous structures composed mainly of actin, vimentin, and tubulin. Signaling from cell surface receptors and migration are mediated by rapid assembly of actin filaments predominantly at the plasma membrane or cortical cytoskeleton. The assembly of cytoskeletal network is regulated by multiple classes of actin binding proteins that initiate, polymerize, sever, depolymerize, and terminate filament formation [78]. The interaction between cytoskeletal binding proteins and fibers is often transient and of low affinity. Recently, alterations in regulators of cortical actin cytoskeletal proteins have been implicated in immune deficiency and autoimmune diseases [79]. 

## 10. Wiskott-Aldrich Syndrome Protein (WASP): WASP-Interacting Protein (WIP) Binding

The importance of actin-mediated cytoskeletal regulation in humans is exemplified by Wiskott-Aldrich syndrome (WAS), an immune deficiency disease characterized by recurrent infections, eczema, thrombocytopenia, and an increased risk of autoimmunity and malignancy as a result of abnormal lymphocyte activation. It is an X-linked disease caused by mutations in WASP, a member of actin regulators that function as scaffolds transducing a wide range of signals between proteins or from proteins to membranes to mediate dynamic changes in the actin cytoskeleton [80]. 

WASP functions in multiple cellular processes in immune responses. It links the T-cell receptor (TCR)/CD3 complex to the actin cytoskeleton, enhancing the efficiency of the immunological synapse formation and cytokine secretion [81]. It also promotes homeostasis of regulatory T-cells and controls T-cell activation and effector functions [80, 82]. Most WASP molecules in the cytoplasm of T cells are associated with WASP-interacting protein/WIP, which acts as a chaperone to localize WASP to areas of active actin polymerization including the immunological synapse [83, 84]. Absence of either WASP or WIP induces impaired T cell proliferation in response to TCR/CD3 ligation as well as defective T-cell homing. Importantly, WASP levels can be restored to normal by expressing WIP in WIP-deficient cells suggesting that the WIP stabilizes and regulates the absolute cellular levels of WASP [85, 86]. 

The WIP has also been shown to affect T-cell activation independent of WASP [86]. In resting cells, WIP remains in a complex with WASP and an adapter protein, CrkL [83]. In activated T cells, the CrkL interacts with phosphorylated ZAP-70, a critical adapter molecule near the cell membrane and recruits the WASP-WIP-CrKL complex to the immunological synapse [87]. At the synapse, WIP is phosphorylated by PKC*θ*, resulting in the release of WASP which upon activation by the membrane-bound Cdc42 kinase initiates actin polymerization. Free WIP binds to newly formed actin filaments and helps stabilize the immunological synapse [82, 86]. Furthermore, WIP regulates the activity of the NF-AT/AP-1 transcription factor complex. AP-1 is a heterodimer of Fos and Jun proteins that both regulate the transcription of multiple biological mediators including the cell surface receptors and directly facilitate the entry of T cells into cell cycle. WIP overexpression is associated with increased actin stabilization and enhanced AP-1 in activated T cells [88]. 

Structurally, WASP proteins are multidomain proteins consisting of a conserved enabled/VASP homology-1 (EVH1) domain, also referred as the WASP homology 1 (WH1) domain, a GTPase binding domain, a proline-rich region, and a basic motif connected through a central region to a WH-2 motif that binds the actin nucleating complex [80, 82]. Most missense mutations in WAS involve the residues in the EVH1 domain of WASP [80]. EVH1 domains have been found in ~630 human genes and are classified into four distinct protein families based on amino acid sequence analysis that includes WASP; enabled/vasodilator-stimulated phosphoprotein (Ena/VASP); Homer/Vesl; sprout-related proteins with an EVH1 domain (SPRED). Each EVH1 subclass recognizes a distinct pattern of amino acids, but all of them bind proline-rich sequence in the left-handed PP_II_ conformation. Residues flanking the PRM contribute to the binding specificity of the EVH1 complexes [12]. 

**Figure 2:**
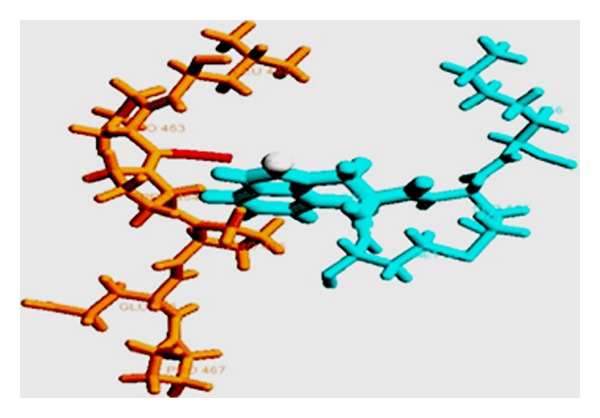
PDB 1MKE depicting the solution structure of WASP:WIP complex, the PRM is labeled.

The primary structure of WIP consists of a highly conserved verprolin homology (VH) domain that binds actin filaments, multiple putative SH3-binding domains for interacting with adapter/signaling molecules and a WASP-binding domain (WBD) [86, 88]. The WBD of WIP is approximately 30 residues long with a highly conserved central proline-rich motif and two short epitopes on either side of the motif. Nuclear magnetic resonance studies and glutathione S-transferase pull-down assays have demonstrated that the conserved polyproline motif of WIP occupies the canonical binding site in the WH1/EVH1 domain of WASP. The WIP polyproline motif forms a PP_II_ helical turn and straddles the highly conserved tryptophan side chain of WASP at the WH1 domain interface, the binding contributing nearly 40% of the total buried surface of the WIP-EVH1 complex [82, 83, 86, 88]. Secondary structure analysis of the WBD of WIP by PROSS showed that the residues exhibited *ϕ* and Ψ angles of −65.9 and 157.3, respectively, consistent with PP_II_ helical conformation [70] (Tables 1 and 2). Interestingly, the WIP polyproline motif has been shown to bind WASP in the opposite direction through an elongated WBD as compared with other EVH1:peptide complexes [92] (Figure 2). The interactions between the conserved phenylalanine residues in the epitope preceding the polyproline motif and the hydrophobic surface of the WH1 domain of WASP (Val, Ala) as well as the formation of a salt bridge between the conserved glutamine in the binding pocket of the WH1 domain of WASP and the acidic residue (K/R) localized in an epitope following the polyproline motif of WIP have been shown to facilitate the reverse orientation [83, 92]. Mutation of WASP residues involved in interaction with any of the three WIP epitopes reduces the WASP binding. These structural features support the observations that different missense mutations disrupt the intermolecular interactions and accelerate degradation of WASP similarly in WAS patients with different genotypes. Thus, the WIP/WASP structure exhibits semi-independent composite linear motifs that are recognized in an extended conformation with enhanced specificity [12, 80, 86]. A similar example of a scaffolding interaction with bidirectional binding of composite linear motifs has been reported for complexes that regulate mitogen-activated protein kinase pathways [12, 92]. 

Recently, it has been reported that treatment with a peptide derived from the proline-rich WBD of WIP restored WASP to physiological levels in lymphocytes from patients with mutations in the WBD of WASP. Furthermore, treatment with the WIP peptide ameliorated the defects in the reorganization of actin cytoskeleton in T cells from these patients [93] (Figure 2). 

## 11. PRM:PRD Interactions in Transcriptional Regulation

Eukaryotic gene expression is a dynamic process regulated by multiple signaling networks mediated by rapid and reversible PPI complexes. Formation of ternary complexes of transcriptional regulatory proteins is critical for gene transcription. These complexes include cross-talk between different families of transcription factors and the interactions of transcription factors with coactivators or corepressors. Transcription initiation requires the formation of an initiation complex that consists of RNA polymerase II, the basal transcription machinery (made up of TFIIA, TFIIB, TFIID, TFIIE, TFIIF, and TFIIH), and the sequence-specific promoter binding transcription factors [94]. Many transcription factors mediate transcriptional activation by interacting through their transactivation domain with one or more components of the basal transcription machinery. Such direct interactions are thought to bring the activation domain over large distances into close proximity with the initiation complex close to the transcription start site [95]. In this context, it is interesting to note that PRMs have been frequently observed in many transcription factors suggesting that the flexibility offered by such segments potentially contribute to the interactions involved in the formation of functional multiprotein transcriptional complex [96].

## 12. PRM in p300:p53 Interactions

The p300 protein is a versatile coactivator with several conserved domains including the bromodomain which recognizes acetylated residues; cysteine-histidine-(CH-) rich domains; a KIX domain; an ADA2 homology domain. While the amino and carboxy termini of p300 activate transcription, the histone acetyltransferase activity is mediated by the central region. The modular organization of p300 provides a scaffold for assembly of multicomponent coactivator complexes that regulate transcription through multiple mechanisms. These include providing a scaffold for recruiting many transcription factors, acting as a bridge to connect sequence specific transcription factors to the basal transcription apparatus, mediating complete activation of select transcription factors via an intrinsic histone acetylase activity, as well as influencing chromatin activity by modulating nucleosomal histones [97]. Interestingly, phage-peptide display analysis suggested that the p300 protein exhibits a strong affinity to bind proline rich peptides [98]. 

The p53 is one of the most well-studied eukaryotic transcription factors that functions as a homotetramer. It is upregulated in response to cellular stress and induces up- or downregulation of genes involved in cell cycle arrest, DNA repair, apoptosis, antiangiogenesis, and senescence pathways [99]. Structurally, it has a modular domain architecture consisting of independently folded DNA-binding domain and tetramerization domains flanked by natively unfolded regions in the amino and carboxy termini. Activation of p53 is associated with various posttranslational modifications of multiple lysine residues at the carboxyl terminus. The amino terminus consists of an acidic transactivation domain (TAD) including a proline-rich region. The TAD binds many components of the transcription machinery including the coactivators p300/cAMP-response element binding protein (CREB), binding protein (CBP), as well as the negative regulators MDM-2 [99, 100]. The binding with p300 is essential for the transcriptional function of p53 [98]. 

The p300 binds the carboxy terminal regulatory domain of p53 predominantly via its bromodomain [99]. This docking releases the p53 of its intrinsic conformational constraints, allowing phosphorylation of critical threonine and serine residues in the activation domain, thus facilitating stabilization. Additionally, the TAD at the amino terminus of p300 interacts with the proline-rich “PXXP” motifs of the p53 activation domain [99, 100]. The binding of the amino and carboxy termini of the p300 with the transactivation regulatory regions of p53 induces a conformational alteration that promotes the sequence-specific DNA binding of p53. The acetylation of critical lysine residues of p53 by the histone acetyltransferase activity of p300 then promotes the transcriptional activity. This concomitant binding of p300 to a relatively ubiquitous proline repeat motif and the classic hydrophobic LXXLL motif of the p53 terminal regions highlights an additional layer of combinatorial regulation of the core p300 protein-protein interactions at a promoter region [98, 101]. Chromatin immunoprecipitation studies showed that the deletion of the proline repeat motif of p53 prevents DNA-dependent acetylation of p53 by occluding p300 from the p53-DNA complex [98]. Although the pathological role of p53 in many neoplasms has been well characterized, the mechanisms of DNA damage and the contribution of abnormal p53 in autoimmune inflammatory diseases such as ulcerative colitis and rheumatoid arthritis are recently recognized. Intriguingly, peptides derived from the “PXXP” containing proline repeat domain of p53 have been shown to bind p300 and inhibit sequence-specific DNA-dependent acetylation of p53 [98].

## 13. Glucocorticoid-Induced Leucine Zipper:p65 Interaction

The mammalian NF-*κ*B family of inducible transcription factors is responsible for regulating specific sets of genes in many cell types and participates in many cellular processes, including inflammation, proliferation, and cell survival. The most common form of NF-*κ*B is a p65:p50 heterodimer, which in resting T cells remains in the cytoplasm as an inactive complex bound to the I*κ*B inhibitor proteins. Following T-cell activation, degradation of I*κ*B releases NF-*κ*B, allowing the subunits to translocate to the nucleus [102]. The ability of p65 to recruit the histone acetyltransferase activity-associated complex consisting of p300 and other coactivators within the nucleus governs the transcriptional regulation. The p300 induces acetylation of a critical lysine residue in the rel homology domain of p65 which then binds to specific sites in the promoter regions of target DNA elements and transiently activates transcription of proteins involved in immune or inflammatory responses and cell growth control [103]. Misregulation of NF-*κ*B is linked to a wide variety of human diseases including infections, inflammatory autoimmune disorders, and various cancers. Hence, specific inhibitors of this nuclear factor are being sought and tested as treatments [104]. 

Glucocorticoids are well characterized anti-inflammatory and immunosuppressive agents. Glucocorticoids act by binding the glucocorticoid receptor in the cytoplasm, thus activating the receptor, which then translocates to the nucleus where it suppresses p65 acetylation by competing with the p300 histone acetyltransferase activity [105]. In addition, the intranuclear glucocorticoid receptor binds specific negative and positive glucocorticoid response elements in target DNA to directly suppress immune response and to activate transcription of anti-inflammatory genes [106]. 

The glucocorticoid-induced leucine zipper (GILZ) was recently identified during a systematic study of genes transcriptionally induced by glucocorticoids [107]. Expression of GILZ is downregulated following T-cell activation [108]. Blockade of T-cell activation either by interfering with T-cell costimulatory molecules or by blocking intracellular signaling pathway has been shown to upregulate GILZ [74, 108]. In addition, treatment with exogenous GILZ has been shown to suppress inflammatory responses [109–111]. Mechanistically, GILZ-mediated effects on immune and inflammatory responses have been attributed to its ability to inhibit NF-*κ*B activation [112]. 

**Figure 3:**
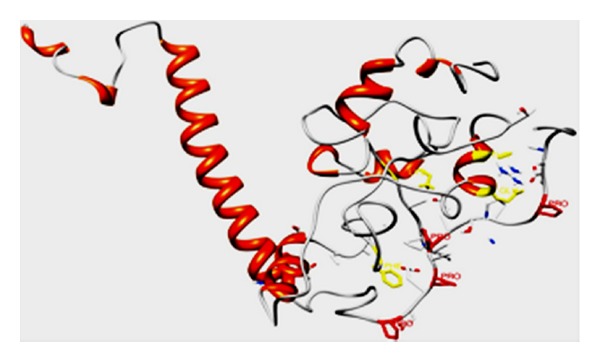
The GILZ:p65 complex: homology modeling of GILZ and p65 was predicted using the structures of delta sleep inducing peptide (PDB:1DIP) and (PDB: 2IW3), respectively, as templates by Geno 3D [89] and SWISS model [90]. Prediction of the structural complex was performed by ClusPro [91]; the complex with least energy is shown.

GILZ has been shown to physically bind the p65 subunit of NF-*κ*B through a protein-protein interaction [113]. Since the interaction is independent of the rel-homology domain and the phosphorylation of inhibitory proteins, it has been suggested that the GILZ binds the transactivation domain of the p65 molecule. Analyses of structural complexes of interactions wherein the binding depends on the presence of one or more prolines have shown that the functionally critical proline/s in the interface of one protein often are in contact with aromatic residues from the other component [114]. In this context, it is interesting to observe that p65-transactivation domain that potentially interacts with the GILZ-COOH presents two highly conserved aromatic residues, F^534^ and F^542^, which are critical residues for p65 transactivation [115]. Structure prediction of p65 and GILZ were generated by homology modeling using Geno3D and Swiss model protein structure prediction servers [89, 90] (data not shown). Docking of p65 with GILZ suggested that the Pro-120 of GILZ was within 5 Å distance of F^534^ of p65 TAD [91] (Figure 3). 

The primary sequence of GILZ consists of an amino terminal leucine-zipper motif and a proline-rich carboxy terminus. Mutational analysis localized the site of interaction with the p65 to the proline-rich carboxy terminus of GILZ (GILZ-COOH). The GILZ-COOH consists of three consecutive (PXX) motifs with a proline as every third residue [110]. Secondary structure assignment based on backbone dihedral angles by PROSS showed that the Pro^120^ of GILZ exhibited a *ϕ* angle of −67° ± 5° and a Ψ angle of 142.5° ± 15° (Tables 1 and 2), thus adopting a PP_II_ helical conformation [70]. Additionally, the presence of multiple glutamic acid residues in the region increases the net charge further promoting the extended conformation by electrostatic repulsion [116]. Recently, a small peptide mimic of GILZ, GILZ-P, has been developed by conceptually integrating the mechanism of action glucocorticoids and the knowledge derived from the structural analysis of GILZ and its interaction with the p65 subunit of NF-*κ*B. Treatment with GILZ-P suppressed T-cell activation and inflammation in a mouse model for multiple sclerosis [117]. It is speculated that the low-molecular-weight GILZ-P can provide promising leads for developing small molecule NF-*κ*B inhibitors.

## 14. Silencing Mediator for Retinoic and Thyroid Hormone Receptors (SMRT) and p65 Interaction

As stated above, the regulated activation and repression of transcription are critical in many biological processes. Controlled repression of transcription is observed in cell-fate decisions during development and cellular differentiation, as well as in the maintenance of homeostasis [94]. SMRT and nuclear receptor corepressor (NCoR) are large homologous corepressor proteins that mediate transcriptional repression by many different nuclear receptors [118]. SMRT and NCoR are also recruited by many other DNA-binding transcription factors, such as BCL6, Kaiso, ETO, MEF2C, CNOT2, and CBF1. Mechanistically, SMRT and NcOR complexes associate with histone deacetylase (HDAC) enzymes and mediate transcriptional repression through deacetylation and condensation of chromatin [119]. 

Consistent with observation that the acetylation of p65 governs transcriptional activation, the components of a corepressor complex for NF-*κ*B include HDAC, SMRT, and NCoR [120]. SMRT and NCoR do not exhibit an enzymatic activity but trigger the catalytic activity of HDAC. The SMRT-HDAC complex is responsible for basal repression of classical NF-*κ*B-regulated gene targets in the unstimulated state [121]. In resting cells, NF-*κ*B remains in the cytoplasm complexed with the IKB*α* and IKB*β* heterodimer. Following stimulation, the I*κ*B kinase-*α* (IKK) and IKK*β* mediate phosphorylation of the I*κ*B proteins and release the NF-*κ*B subunits for nuclear translocation [102]. The IKK*α* phosphorylates SMRT and initiates derepression, thus preventing HDAC chromatin association. This allows the active p50-RelA/p65 of NF-*κ*B to bind DNA and potentiate transcription [118, 119, 122]. Thus, the SMRT-dependent transcriptional regulation of NF-*κ*B plays a critical role in controlling cellular proliferation. 

**Figure 4:**
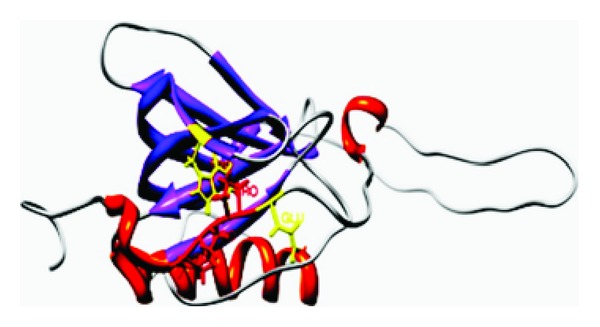
SMRT: p65 complex: the SMRT structure was derived from the Chain A of the PDB 2ODD. Homology model of p65 was predicted using the structures of PDB:2IW3 as templates by Geno 3D by Geno 3D [89] and SWISS MODEL [90]. Prediction of the structural complex was performed by ClusPro [91], the complex with least energy is shown.

Structurally mammalian SMRT has two amino terminal SNT (Swi/Ada/N-CoR/TFIID)/DNA-binding domains and two receptor interaction domains that present corepressor nuclear receptor (CoRNR) motif near the carboxy terminus [119, 121]. In addition, SMRT also contains three repression domains (RDs) that recruit diverse proteins. The third RD domain of SMRT includes proline-rich regions and has been shown to mediate transcriptional regulation by interacting with the ligand-activated glucocorticoid receptor [120]. Yeast two hybrid system and glutathione S transferase pull-down assays showed that the residues encompassing the proline rich SMRT RD3 region specifically and selectively bind the residues of the transactivation domain of p65. Significantly treatment with SMRT peptide derived from this proline-rich region has been shown to physically interact with the p65 and inhibit transactivation of inflammatory proteins via recruitment of HDACs [123] (Figure 4). 

## 15. MYND:PRM Interactions

The MYND domain is a zinc-binding domain present in a large number of proteins that participate in many protein-protein interactions involved in transcriptional regulation. Some of the proteins with MYND domain include BS69, a transcriptional corepressor; the chimeric fusion protein of acute myelogenous leukemia (AML) and ETO (a nuclear protein that interacts with corepressor molecules) (AML-1-ETO); the bone morphogenesis protein receptor-associated molecule 1 (BRAM1), deformed epidermal autoregulatory factor-1 (DEAF-1), and SET and MYND domain-containing proteins (SMYD) [124–126]. Functionally, many MYND domain containing proteins have been involved in diverse cellular processes including proliferation, apoptosis, adhesion, and migration. Although the role of most MYND domains has been investigated with respect to tumorigenesis, their role in hematopoietic development suggests a potential role in normal immune response as well as in immunopathology [127, 128]. 

**Figure 5:**
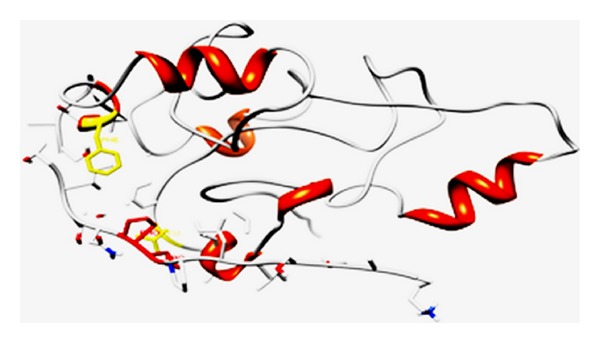
The SMRT:MYND complex (PDB 2ODD) with proline in the interface highlighted.

The MYND domain typically recognizes proline-rich motifs in partner proteins. For example, molecular studies have shown that the BS69 MYND binds the viral oncoproteins EA1 and EBNA1 as well as the Myc-related cellular transcription factor (MGA) through “PXLXP” motif conserved in all three interacting partners [124]. The chimeric AML1-ETO protein contains the DNA-binding domain of AML1 and nearly all of ETO [129]. The ETO hosts a MYND domain at the carboxy terminus and has been shown to physically associate with N-CoR/SMRT and their associated HDACs to aberrantly repress transcription [130]. Mutational studies suggested that the proline-rich motif “PPPLI” in the SMRT-RD3 specifically interacts with the ETO MYND domain [131]. Interestingly, a peptide derived from the SMRT-RD3 has been shown to specifically bind the MYND domain of AML/ETO. Solution structure of the MYND-SMRT peptide complex suggested that the SMRT PRM binds in an extended conformation to a hydrophobic pocket in MYND [130] (Figure 5). The *ϕ* and Ψ angles of the critical proline residues are consistent with PP_II_ helical conformation (Tables 1 and 2) [70]. The side chain of the critical proline in the SMRT PRM is packed on top of a highly conserved tryptophan in the ligand-binding region of the MYND domain. The carbonyls of the second and third prolines of the SMRT PRM form hydrogen bonds with the conserved glutamine and serine residues of the MYND domain at the binding interface. Although hydrogen bonds to the backbone generally cannot provide specificity, the relative geometrical positions of the highly conserved tryptophan, glutamine, and serine in MYND-binding domain are thought to favor interaction with the elongated conformation of the SMRT/NcOR PRM [130]. 

Since transcriptional regulation involves direct interactions between the transactivation domains of a transcription factor with either coactivators/corepressors in the transcriptional machinery to initiate or suppress transcription, molecules that directly block the formation of these complexes would then function as transcriptional modulators [132]. Peptides or small molecule mimics of the transactivation domain of transcription factors should be able to competitively interfere with its natural counterpart. However, translation of this concept has been highly challenging as evidenced by the few synthetic transactivation domain inhibitors reported in recent years.

## 16. Conclusion

Increasing knowledge of the interactome in the physiological and pathological immune responses provides an unprecedented opportunity for identification and characterization of potential diagnostic and therapeutic targets. Although, it is recognized that protein-protein interaction interfaces may be dissected into much smaller contact points, and only a small number of amino acids are critical to the specificity of the interactions, comprehensive rules are still difficult to derive [133]. Despite this, an often used strategy in the discovery of peptide drugs is an exploitation of the complementary surfaces of naturally occurring binding partners. It is expected that these peptides function as competitive inhibitors, masking an interaction site and making it inaccessible for the binding of the protein from which it has been derived. The inhibitory peptides could serve as potential drugs by themselves, and also more importantly, knowledge about the structure of the critical amino acids at the interface could be used as a basis to design a collection of potential mimetics [134].

## Figures and Tables

**Table 1:** The dihedral angles of the proline-rich motifs in selected immune-response-related proteins.

Class			Motif	Critical proline	
Cell surface proteins	**CD152**			Phi	Psi
	Mouse	1DQT	MYPPPY	−84	167
	Human	1I8L	MYPPPY	−76	164.6
	**CD28**				
	Mouse	Model	MYPPPY	−53.4	166.5
	Human		MYPPPY	−55.4	165.3
	**ICOS**				
	Mouse	Model	FDPPP	−53.4	167.4
	Human		FDPPP	−83.9	124.2

Cytoskeletal proteins	**WIP**	2IFS	LPPP	−65.9	157.3
		1MKE	LPPP	−75	158.1

Transcriptional factors	**GILZ**				
	Human	Molecular Model	PEAP	−67.5	142.5
	Mouse		PEAP	−72.5	162.3
	**SMRT**				
	Human	2ODD	PPP	−70	158.4

**Table 2:** The proline-rich motif (PRM) and the proline-rich motif binding (PRB) domain in immune-related proteins.

	Protein receptor	PRM	PRM interactant	PRD critical residue	Evidence
Membrane associated	CD28	MYPPPY	CD80	Y71	Mutagenesis
	CD152	MYPPPY	CD86	F	Mutagenesis, structural analysis
	ICOS	FDPPP	ICOSL	Y53	Mutagenesis, molecular model

Cytoplasmic	WIP	LPPP	WASP	WHI1 domain, W54	Structural analysis

Signal transduction	GILZ	PXX	p65	TAD (F534. F542)	Immunoprecipitation

Transcriptional cofactors	p53	PXXP	p300	(SPC1 192–337) SPC-2 (1737–1913)	ChIP
	SMRT	PXLXP	p65	TAD	GST pull-down assays
	SMRT		MCTF	MYND domain	Mutagenesis
			EA1	MYND domain	Solution structure
			ETO	MYND domain	Solution structure

Myc-related cellular transcription factor; viral oncoproteins EA1; ETO (a nuclear corepressor protein) chromatin immunoprecipitation.
